# Geographical spread of gastrointestinal tract cancer incidence in the Caspian Sea region of Iran: Spatial analysis of cancer registry data

**DOI:** 10.1186/1471-2407-8-137

**Published:** 2008-05-14

**Authors:** Mohammadreza Mohebbi, Mahmood Mahmoodi, Rory Wolfe, Keramat Nourijelyani, Kazem Mohammad, Hojjat Zeraati, Akbar Fotouhi

**Affiliations:** 1 Department of Epidemiology and Biostatistics, Tehran University of Medical Sciences, Tehran, Iran; 2 Department of Epidemiology and Preventive Medicine, Monash University, Melbourne, Australia

## Abstract

**Background:**

High incidence rates of gastrointestinal tract cancers have been reported in the Caspian region of Iran. This study aimed to: 1) describe the geographical spatial patterns of gastrointestinal tract cancer incidence based on cancer registry data and, 2) determine whether geographical clusters of statistical significance exist.

**Methods:**

The Babol Cancer Registry, which covers the two major northern Iranian provinces of Mazandaran and Golestan (total population = 4,484,622) was used to identify new gastrointestinal tract cancer cases during 2001 to 2005. Age-specific cancer incidence rates were calculated for 7 gastrointestinal tract cancer sites in 26 wards of the Mazandaran and Golestan provinces. Spatial autocorrelation indices, hierarchical Bayesian Poisson models, and spatial scan statistics were used in measuring the geographic pattern and clusters.

**Results:**

There were non-random spatial patterns in esophageal and stomach cancers that were similar for both sexes. Clusters of high incidence were identified in esophageal, stomach, colorectal and liver cancer for both sexes, as well as a possible cluster of pancreas cancer in males.

**Conclusion:**

Gastrointestinal tract cancers exhibit significant spatial clustering of risk in northern Iran. Further work is needed to relate these geographical patterns to information on potential life-style and environmental factors.

## Background

Approximately 50,000 new cases of cancer occur each year in the Iranian population of 70.4 million. The most common organ system involved with more than 38% of all cancers is the gastrointestinal (GI) tract. Stomach, esophagus, and colorectal are the three most common cancers in males; in females, after breast cancer, esophagus, stomach, and colorectal are the major cancers [1,2]. Cancer is the third most common cause of death in Iran, accounting for 14% of mortality. Overall, GI cancers account for nearly half (44.4%) of all cancer related deaths in Iran. Unfortunately, GI cancers often come to medical attention when they are at advanced stages and so limited or no effective therapies are available to treat them [3,4]. Theoretically, these cancers may be treatable in their early stage; therefore early detection is desirable.

A cancer registry maintained by the International Agency for Research on Cancer (IARC) and the Institute of Public Health Research of Tehran University showed that from June 1968 to June 1971 the age-adjusted incidence rates of esophageal cancer for both males and females from the north-eastern part of the Golestan province were more than 100 per 100 thousand persons per year and were among the highest rates in the world [5-9]. There was evidence of sharp gradients in incidence rates over relatively short geographical distances. Rates appeared to decrease moving westward for some 400 km along the southern Caspian region where the incidence was approximately one tenth to one fifth that of the Golestan province [5]. Due to the sociopolitical changes in 1979, study of these cancer rates discontinued before complete patterns of incidence and the full complement of risk factors could be established. Until recent years, there was no comprehensive report of incidence rates of cancer in Iran in general and in the Caspian Sea region in particular. Recently results of a population-based cancer survey from Ardabil and Golestan provinces, respectively in the western and eastern parts of the Caspian region, were published and showed significant changes in cancer incidence rates in this region compared to 30 years ago [10,11]. There was evidence of a declining rate in esophageal cancer [11] but an increasing rate of colorectal cancer especially in young (< 40 years) people [12].

To combat this disease, accurate up-to-date epidemiological information is an important weapon. The study of spatial variation in incidence is a vital component of descriptive epidemiology. Many cancer atlases have been used for this purpose, and some studies have used formal statistical analyses of the spatial pattern of the disease [13,14].

The aim of this study was to examine the geographic pattern of gastrointestinal tract cancer incidence in the southern region of the Caspian Sea using data from a new cancer registry and active surveillance conducted by the Institute of Public Health Research of Tehran University of Medical Sciences in the Mazandaran and Golestan provinces of Iran.

The total population of these two provinces is approximately 4.5 million (1.6 million in Golestan province) constituting about 6.4% of the total Iranian population. As with the population of Iran more generally, the population of these two provinces is young: 36% are ≤ 15 years and less than 4% are > 65 years, and 51% of the population live in rural areas. The life expectancy at birth in Mazandaran province is 67.7 years for males and 70.5 for females, and is similar in Golestan [15].

**Figure 1:**
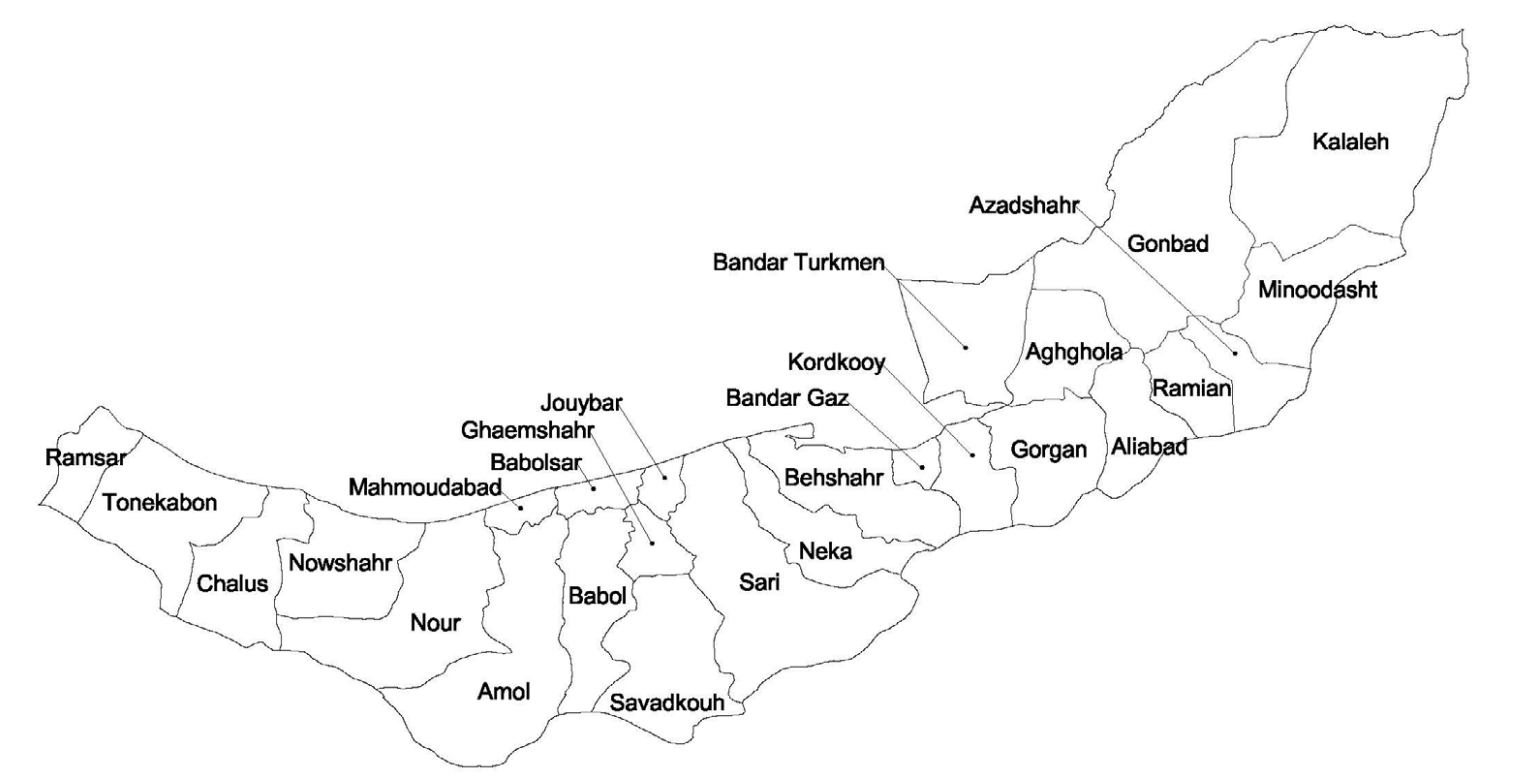
Geographic boundaries of wards in Mazandaran and Golestan provinces in the Caspian Sea region of Iran.

Mazandaran province has an area of about 23,800 km^2^, about 1.5% of the land area of Iran and is located in the north of Iran. Golestan province is located in north east of Iran (east of Mazandaran), south-east of the Caspian Sea, and covers an area of 20,900 km^2^, constituting 1.3% of the country. Currently, there are 15 and 11 wards in Mazandaran and Golestan provinces respectively as shown in Figure 1.

## Methods

### Cancer reporting and Babol cancer registry

The Caspian Cancer Registry located in the city of Babol was established in 1969 by joint collaboration of the Institute of Public Health Research of Tehran University and IARC [16]. Activity of this center was discontinued due to the sociopolitical events of the 1980s in Iran but resumed in 1995 as a local cancer registry, controlled by Tehran University of Medical Sciences.

The major sources of data collection related to cancer in the Babol cancer registry were reports from pathology laboratories, hospitals, and radiology clinics. All 80 diagnostic and treatment centers in the Mazandaran and Golestan provinces cooperated with the Babol cancer registry. The registry consisted of a full time physician, an epidemiologist, a pathologist and a biostatistician. The survey team responsible for collecting reports of new cancer cases went to diagnostic and treatment centers and checked records for cancer cases monthly. Relevant documents were then sent to the registry office in Babol. Patient name, sex, age at the time of diagnosis, place of residence, pathologic diagnosis, and diagnostic methods were collected. Coding of cancer diagnosis samples was based on the international classification of disease for oncology (ICD-O) coding [17] and were done under direct supervision of pathology specialists. The Ethics Committee of Tehran University of Medical Sciences approved these survey methods and the function of the registry.

### Study Population

Residents of Mazandaran and Golestan provinces constitute the study population. The estimated mid year population between 2001 until 2005, stratified for sex, age in five-year intervals, and place of residence (county/city) was obtained from the statistical center of Iran [18,19]. The cases of interest were all digestive system cancer patients registered between 2001 until 2005 among the study population based on ICD-O coding, including C00–C26. Among these esophageal cancer (C15), stomach cancer (C16), small intestine (C17), colon and rectosigmoid cancer (C18, C19), liver (C22), gallbladder (C23), and pancreas (C25) are investigated here.

### Quality of Data Collection

The survey team had access to treatment records of cancer patients and pathology reports indicating a cancer. Most private radiology centers recorded the identifying data of patients and only occasionally kept the radiologic or sonographic reports, while the public centers, usually located in the hospitals, kept radiology reports for the majority of patients. All private and public hospitals and clinics had a filing system for endoscopy reports. All information collected from cooperating centers was checked again at the Babol cancer registry for completeness, for accuracy of demographic information, and was cross-checked with pathological records. Diagnosis of cancer was based on histopathology in 68.2%, clinical or radiology in 29.7% and death certificate only (DCO) in 2.1% of all cancer cases [20]. The DCO information has not been used in our analysis. Percentages of microscopic verification (MV) for some important GI cancer sites are shown in Table 1.

**Table 1:** Percentage of microscopic verification for the most common gastrointestinal tract cancer sites

Site	%
Esophageal	47.7
Stomach	49.6
Colorectal	95.9
Liver	86.4
Bladder	94.7

About 3% of cases lacked residential information at county/city level but this ranged from about 1% for cancers with high hospitalization and case-fatality rates (e.g. pancreas) to about 15% for cancers commonly registered only by pathology reports (e.g. melanoma) [20]. In order to use the cases with unknown residential information, the geographic referral pattern for each hospital or diagnosis center was used to assign residences on a proportional as-likely basis.

Reported cancer cases were stored in an Excel data bank. Two independent groups of operators were responsible for entering new cases in separate data banks and these were checked against each other to remove data entry errors. After completion of data entry, all patients were alphabetically ordered and duplicate cases with the same name, sex, age and place of residence were eliminated by manual and computerized searching. A number of residents of Mazandaran and Golestan seek medical care outside the region, mainly in Tehran, and occasionally in neighboring Gilan province. In order to make sure of reasonable coverage, cancer cases recorded by Ramsar Cancer registry (a population based cancer registry conducted by Tehran Medical University for Gilan province) and Cancer Institute of Shariati Hospital (a hospital based cancer registry which is the most reliable and comprehensive cancer data bank in Tehran) were searched to find any cancer cases from Golestan and Mazandaran inhabitants for the same time period (2001–2005).

Concordance of residential place information within one year of diagnosis was examined for patients with multiple records during 1998–2000. For this, the coded place of residence (normally from the earliest source record after diagnosis), was compared with those of up to five later source records. Concordance was generally high, for example, agreement on place of residence between the first diagnosis record and the next was 94% for stomach and 92% for esophageal cancer [20].

### Statistical methods

We calculated the age standardized incidence ratio (SIR) of each ward for each sex. The population of the region was fairly stable between 2001 and 2005 so 2003 figures were used as the standard population, and indirect standardization was used to calculate SIR [21]. In order to compare the incidence rates in the Mazandaran and Golestan region with other parts of the world, directly standardized incidence rates using the 1970 (Segi's World population) [22,23] and 2000 (WHO World Population) [24] standard world population were also calculated.

Two methods were used to measure spatial aggregation of the ward SIRs; Moran's I and Geary's C [25,26]. Both measures are a correlation-type index based on continuous data values, but neither index's scale has an interpretation that corresponds to conventional correlation coefficients which take values in the range (-1, 1). For Geary's ratio, a value of 1 indicates a random pattern of spatial variability in incidence, whereas a value greater than 1 suggests a dispersed pattern with adjacent wards having different incidence, and a value less than 1 suggests a clustered pattern in which adjacent wards have similar incidence. The numeric scale of Moran's I is related to its expected value, E(I), under a random spatial pattern. Values less than E(I) are typically associated with a uniform/dispersed pattern and values greater than E(I) typically indicate a clustered pattern. The criterion of contiguity used for calculating the spatial weights matrix was centroid distance [27].

The SIR are crude estimates of underlying ward-specific relative risks because of sampling uncertainty where they are based on small numbers of cases hence smoothed estimates of these relative risks (RR) were calculated using the autoregressive conditional model [28,29]. We used a spatial Poisson model with two random effect terms for each of the following: a) effects which vary in a structured manner in space (region contiguity); and, b) effects which vary among municipalities in an unstructured manner (region heterogeneity). The model takes the following form

O_i _∝ Poisson(E_i_λ_i_)

log(λ_i_) = α + h_i _+ b_i_

where: λ_i _is the relative risk in area i; O_i _is the number of deaths in area i; E_i _are the expected number of cases calculated from indirect standardization; α is the intercept; h_i _is the municipal heterogeneity term; and b_i _is the spatial term. We used a non-informative Normal prior distribution for b_i _and a conditional autoregressive (CAR) prior distribution for h_i_[28]. The criterion of contiguity used was ward adjacency.

The models were fitted using Markov Chain Monte Carlo (MCMC) simulation methods with improper priors [30]. The Bayesian statistical software (WINBUGS 1.4) was used for computing two independent Markov Chains [31]. Initial values of all stochastic nodes of the model were chosen to provide dispersed initial values without being excessively over-dispersed. For the common intercept, α, and random effects h_i_, and b_i _zero (0) was used to initiate one chain, and estimated parameter values from a Poisson model fitted to all GI cancers for both sexes were used to initiate the other chain. After a burn-in of 50,000 iterations, the following 450,000–650,000 iterations were sampled from each of the two chains choosing every tenth iteration to avoid possible autocorrelation. This large sample approximation of the stationary posterior distribution for each ward relative risk was summarized in WINBUGS and was subsequently transferred into Geographic Information System software for mapping.

We used the spatial scan statistic [32] to detect local clusters in smoothed RR maps. This test has been shown to have good power for detecting localized hot-spots of excess events [33,34]. The statistic is defined by imposing circular windows with variable radii ranging from zero to a user defined upper bound on the map. The base of the window is in turn centered on each of several possible centroids. We used SatScan software [35] for this purpose in which, for each circle, the log likelihood ratio was calculated and the p-values obtained by a Monte Carlo simulation procedure.

In the present study, for each location and size of the scanned space in the area under study, the alternative hypothesis refers to elevated smoothed RR inside the space as compared to outside, and a p-value less than 0.05 was used for statistical significance. The scan was set at a maximum spatial cluster size of 25% of the population under study.

### Cartographic display

**Figure 2:**
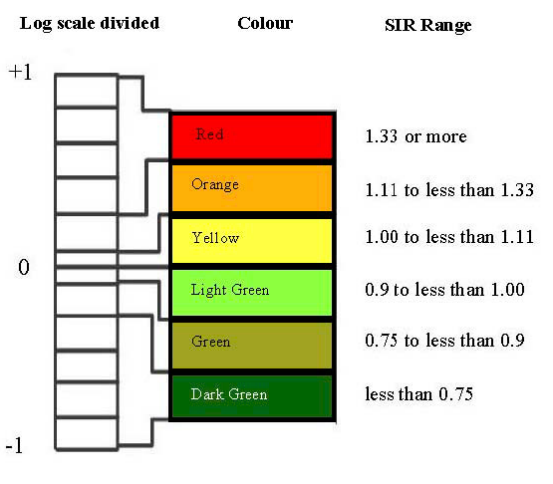
Relative risk (RR) categories.

In this study the RR break points were determined by considering values in the range 0.1 to 10. This corresponds to the range -1 to +1 upon logarithmic transformation. Then this logarithmic scale was divided into 11 equal intervals centered on zero, the break point values were transformed back to the original RR scale, and the five middle intervals were used in the maps. As shown in Figure 2, the middle category was further divided above and below 1. A red-green color scheme was used for the maps, with shading of red for areas with the highest RR, followed by orange and yellow for areas with moderately elevated RR, light and medium green for areas with moderately low RR, and dark green representing areas with the lowest RR. These maps were redrawn and augmented by highlighting the most likely clusters with dots and secondary clusters with slashes.

## Results

A total of 5826 new GI cancer cases were diagnosed in 2001–2005 in Mazandaran and Golestan. Of these, 27 cases were diagnosed with rare GI sites (anus and anal canal (C21), and other unspecified parts of the biliary tract (C24), or other ill- defined digestive organ (C26)) and were excluded from our spatial analysis. Of 5799 remaining cases, 3504 (60.4%) were male. Table 2 shows incidence rates, number of cases and autocorrelation indices for GI cancers by site of the cancer and sex. As expected, the smoothed ward-specific RR had less variation than the SIR, e.g. 5^th ^to 95^th ^percentiles for RR were 0.48 to 1.68 and for SIR were 0 to 2.14.

**Table 2:** Incidence rate and directly standardized incidence rates (per 100000 person-years) using the 1970 and 2000 world population of GI cancers 2001–2005 in Mazandaran and Golestan provinces of Iran and indices of spatial autocorrelation by cancer site and sex

Cancer Site/Type	Sex	No. of Cases	Incidence Rate	1970 world population	2000 world population	Moran's I^#^	Moran's I p-value	Geary's C	Geary's C p-value	Type of spatial pattern
Esophagus	M	891	8.10	12.16	14.61	0.016	0.07	0.78	0.04	clustered
	F	810	7.23	11.27	12.73	0.021	0.06	0.77	0.04	clustered
Stomach	M	1838	15.62	23.04	26.78	0.130	0.04	0.86	0.06	clustered
	F	827	6.46	9.92	11.25	0.059	0.06	0.88	0.06	clustered
Colorectal	M	556	4.88	6.74	7.55	-0.182	0.08	1.17	0.08	dispersed
	F	478	4.32	6.25	6.86	-0.146	0.07	1.14	0.09	dispersed
Gallbladder	M	30	0.27	0.21	0.39	-0.033	0.36	0.90	0.24	inconclusive or random
	F	62	0.56	0.51	0.89	-0.036	0.28	1.01	0.32	inconclusive or random
Pancreas	M	43	0.39	0.35	0.55	-0.014	0.41	0.89	0.25	inconclusive or random
	F	24	0.22	0.21	0.34	0.014	0.33	0.93	0.31	inconclusive or random
Liver	M	103	0.94	0.93	1.49	-0.050	0.11	1.21	0.07	inconclusive or random
	F	63	0.57	0.58	0.97	-0.053	0.12	1.11	0.90	inconclusive or random
Small intestine	M	43	0.31	0.44	0.48	-0.004	0.23	1.06	0.34	inconclusive or random
	F	31	0.23	0.34	0.36	-0.025	0.25	0.93	0.35	inconclusive or random

^# ^E(I) for all tests are -0.04

**Figure 3:**
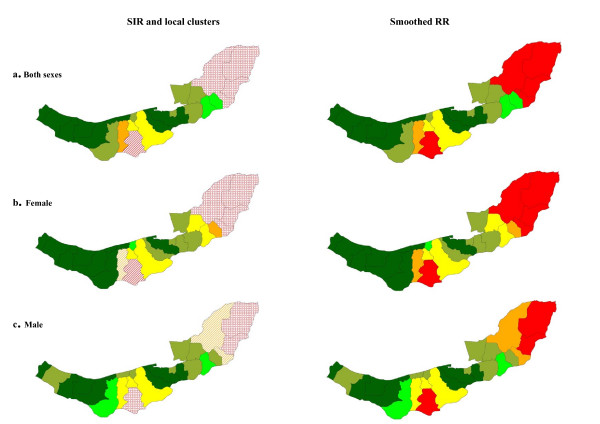
Spatial pattern, local clusters (wards shaded with "dots" indicate the most likely clusters, and wards shaded with "slashes" indicate the secondary clusters), and smoothed RR of esophageal cancer incidence.

Figure 3 shows strong spatial aggregations in esophageal cancer for both males and females with a tendency for high rates in eastern and central wards and low rates in the west. The significance of these overall spatial trends were supported by Moran's I and Geary's C indices (Table 2). Two local clusters were detected in each sex, details of their location and characteristics are provided in Table 3 and displayed in the left-hand panels of Figure 3. The clusters were very similar for males and females.

**Table 3:** Spatial scan statistics for detecting local clusters in smoothed RR's

Cancer site	Cluster type*	p-value	No. Cases	No. Expected	Mean inside**	Mean outside**	Location
Esophageal; Both Sexes	M	0.001	459	257.0	1.74	0.82	Azadshahr, Gonbad, Kolaleh, Minoodasht
	S	0.001	147	107.3	1.51	0.94	Savadkouh
Esophageal; Male	M	0.001	227	137.9	1.59	0.81	Azadshahr, Gonbad, Kolaleh, Minoodasht
	S	0.001	24	13.7	1.41	0.91	Savadkouh
Esophageal; Female	M	0.001	232	119.1	1.83	0.84	Azadshahr, Gonbad, Kolaleh, Minoodasht
	S	0.001	123	93.6	1.29	0.97	Babol, Savadkouh
Stomach; Both Sexes	M	0.001	1262	1038.6	1.51	0.80	Amol, Babol, Ghaemshahr, Jouybar, Sari, Savadkouh
	S	0.001	333	235.9	1.34	0.93	Gonbad, Minoodasht
	S	0.001	167	136.2	1.20	0.94	Ramsar, Tonekabon
Stomach; Male	M	0.001	951	769.2	1.42	0.79	Amol, Babol, Ghaemshahr, Jouybar, Sari, Savadkouh
	S	0.001	206	168.8	1.17	0.92	Gonbad, Minoodasht
	S	0.001	114	97.2	1.14	0.92	Ramsar, Tonekabon
Stomach; Female	M	0.001	311	269.4	1.64	0.85	Amol, Babol, Ghaemshahr, Jouybar, Sari, Savadkouh
	S	0.001	101	44.5	2.14	0.99	Gonbad
	S	0.001	53	39.0	1.28	1.02	Ramsar, Tonekabon
Colorectal; Both Sexes	M	0.001	80	56.1	1.46	0.85	Ramsar, Tonekabon
	S	0.001	449	350.6	1.18	0.81	Babol, Babolsar, Ghaemshahr, Jouybar, Mahmoudabad, Sari
	S	0.001	147	83.8	1.67	0.87	Gorgan
Colorectal; Male	M	0.001	289	219.9	1.26	0.78	Amol, Babol, Babolsar, Behshahr, Ghaemshahr, Jouybar
	S	0.001	81	44.8	1.65	0.86	Gorgan
	S	0.001	13	8.1	1.38	0.87	Ramsar
Colorectal; Female	M	0.001	45	26.2	1.61	0.85	Ramsar, Tonekabon
	S	0.001	66	39.1	1.53	0.88	Gorgan
	S	0.001	213	173.8	1.16	0.85	Amol, Babol, Ghaemshahr, Sari, Savadkouh
Liver; Both Sexes	M	0.001	12	5.2	1.91	0.84	Gorgan
	S	0.001	16	10.9	1.33	0.84	Ghaemshahr, Sari
	S	0.001	3	0.9	1.17	0.87	Ramsar
Liver; Male	M	0.001	20	8.7	1.68	0.86	Gorgan
	S	0.001	30	18.1	1.28	0.86	Ghaemshahr, Sari
Liver; Female	M	0.001	3	0.9	2.00	1.03	Ramsar
	S	0.001	12	5.2	1.78	1.04	Gorgan
	S	0.001	12	7.7	1.14	1.06	Sari, Savadkouh
Pancreas; Both Sexes	M	0.001	12	7.8	1.95	0.91	Jouybar, Sari
Pancreas; Male	M	0.001	15	5.0	1.21	1.01	Jouybar, Sari

* M = most likely cluster; S = Secondary cluster.

** Mean of smoothed RR in wards inside and outside the circles generated by Sat Scan analysis.

**Figure 4:**
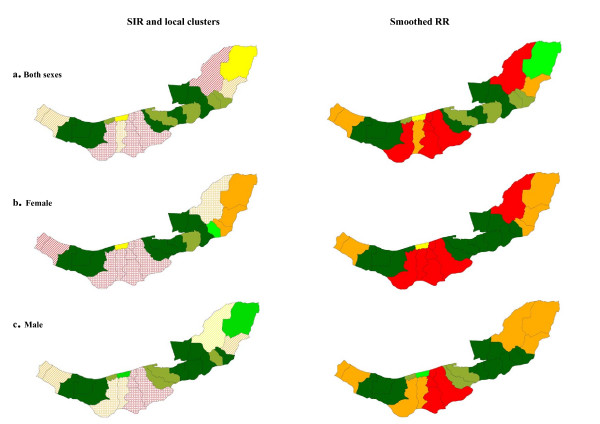
Spatial pattern, local clusters (wards shaded with "dots" indicate the most likely clusters, and wards shaded with "slashes" indicate the secondary clusters), and smoothed RR of stomach cancer.

Strong spatial clustering was found in both sexes for stomach cancer. According to Figure 4, high rates of stomach cancer occurred in central, eastern and western wards. The significant local clusters that were detected are described in Table 3; the clusters were very similar for males and females.

**Figure 5:**
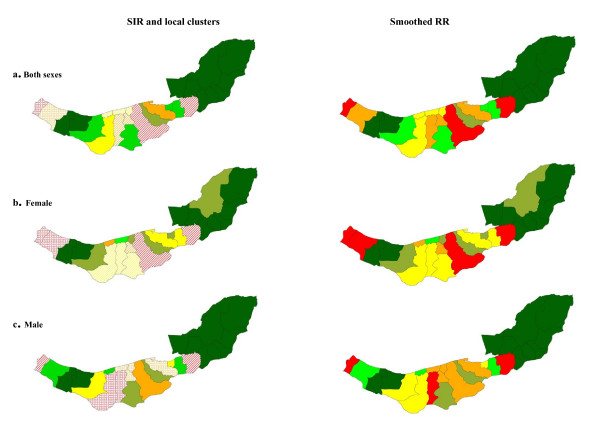
Spatial pattern, local clusters (wards shaded with "dots" indicate the most likely clusters, and wards shaded with "slashes" indicate the secondary clusters), and smoothed RR of colorectal cancer.

The spatial autocorrelation tests in Table 2 did not show evidence of any global spatial pattern for colorectal cancer; this evidence was supported by observed and smoothed maps of SIR. However there was evidence of local clusters on northern and central regions as shown in Figure 5 and described in Table 3.

**Figure 6:**
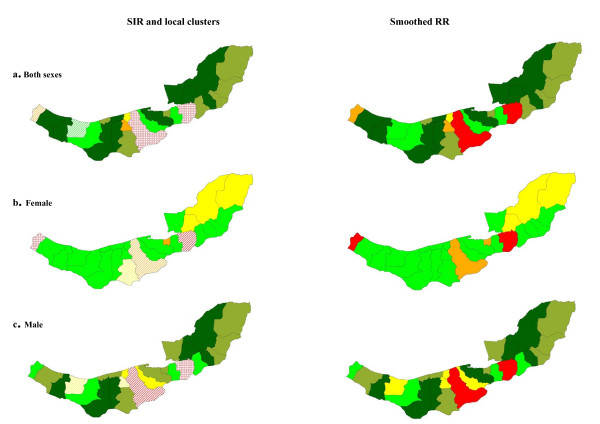
Spatial pattern, local clusters (wards shaded with "dots" indicate the most likely clusters, and wards shaded with "slashes" indicate the secondary clusters), and smoothed RR of liver cancer.

The findings for liver (Figure 6) and pancreatic cancer (Tables 2 and 3) were suggestive of weak or no evidence of spatial correlation although this could be related to the smaller number of cases for these cancers.

## Discussion

Ongoing media reports from non-scientific and scientific commentators on the apparent disease clusters in northern Iran and their possible causes have raised considerable alarm in the region with consequent demands for immediate action.

### Quality of registry data

The overall quality of the registry data was excellent with almost all cancers diagnosed by histopathology or clinical/radiology means. There was a small amount of missing data on place of residence. We consider our use of imputation for cases with unknown residential information to be reasonable because of the small amount of missing data; any bias that may result will be small and in the conservative direction towards accepting the null hypothesis.

### Methodology

Application of Moran's I and Geary's C to public health regional count data merits some thought. Both indices assess the spatial similarity of deviations of each regional count with the overall mean regional count. Due to spatial heterogeneity of regional at-risk population sizes inherent in regional public health data, observed spatial similarity in regional deviations from the mean regional count may simply be due to variations in the regional at risk population size [36]. We adjusted these indices for regional counts by comparing the observed count in each region with its expectation under the constant risk hypothesis, rather than comparing the count to the overall mean count [27,37].

In the MCMC estimation, convergence of relative risk for the two independent chains was confirmed by plotting their traces and observing random mixing of all chains which revealed white noise variation around a common value with no trend. This was supported by observing Brooks-Gelman-Rubin diagnostics that clearly satisfied convergence criteria [38]. As compared with other statistical methods for spatial epidemiology, the spatial scan statistic has the following features that make it particularly suitable as a screening tool for evaluating reported disease clusters: 1) It adjusts for the inhomogeneous population density, 2) By searching for clusters without specifying their size or location, the method ameliorates the problem of pre-selection bias, 3) The likelihood ratio-based test statistic takes multiple testing into account and delivers a single p-value for the test of the null hypothesis, 4) If the null hypothesis is rejected we can specify the approximate location of the cluster that caused the rejection. In addition to the most likely cluster, the method identifies secondary clusters in the data set and can order them according to their likelihood ratio [32].

### Spatial Analysis

#### Esophageal Cancer

Among the investigated GI cancer sites, esophageal cancer is of special interest in this region. Several studies conducted in the 1970's in the Caspian region showed that areas inhabited by Turkmen had a much higher incidence rate of esophageal cancer than those areas with a mainly Persian population, although the differences were less marked within Golestan province [5,9]. Recent studies indicate a declining incidence of esophageal cancer in this region, compared to those reports from 30 years ago. In fact, age adjusted incidence rates of 165.5 per 100 thousand in males and 195.3 per 100 thousand in the 1970's had reduced to 43.4 and 36.3 per 100 thousand respectively for males and females, according to a recent study [11]. Despite this dramatic decrease, the Turkmen plain is still a high risk area. The increasing pattern of SIR from west to east was more systematic in females than in males and there was a secondary cluster in central wards in both sexes. There was a strong significant correlation (0.85) between male and female rates, which supports the notion of a systematic pattern. Tobacco, diet low in fresh fruit and vegetables, and low socioeconomic status (SES) are among major known risk factors for esophageal cancer [39,40]. A case control study in Turkmen regions of Northeastern Iran has suggested that tobacco, alcohol, nass (a drug produced from plant leaves and tobacco), and perhaps opium (two risk factors of potential importance in the area) are not the major etiological factors for esophageal cancer in this region [41]. Esophageal cancer seems to be homogenously distributed among both Turkmen and non-Turkmen, and among both city and village dwellers of Turkmen plain (Gonbad, Minoodasht, and Kalaleh wards in Figure 1), although a familial study in Golestan confirmed a strong familial component to esophageal cancer in the Turkmen population [42]. The dietary patterns in eastern and western wards appear very different and these differences can be explained at least in part by climate and socio-economic differences, however there are no regional data on food consumption patterns in the area to explore this in detail.

#### Stomach Cancer

Stomach cancer shows strong spatial clustering in both males and females, with a significant correlation (0.84) between sexes. Stomach cancer is particularly important because of its relatively high incidence in this region. A large body of evidence supports a causative role for Helicobacter pylori in chronic gastritis [43]. H. pylori infection also increases the risk of stomach cancer [44]. A seroepidemiologic study in different parts of Iran revealed near 90% prevalence of H. pylori infection in adults older than 35 years [45]. Also, a recent study in Ardabil, which is a province in the western part of the Caspian region, revealed nearly 90% H. pylori infection in the healthy population older than 40 years [46].

A diet low in fresh fruits and vegetables, high intake of nitrates/nitrites (e.g., in water and preserved foods), and low SES are other important risk factors for stomach cancer [39]. There was a significant positive correlation between SIR's of esophageal and stomach cancer which may be an indication that these two cancer sites in the region share common risk factors such as smoking, low socio-economic status, low fruit and vegetable intake, and gastric atrophy [47-51].

#### Colorectal Cancer

There was a dispersed pattern for colorectal cancer in both sexes, with a tendency to relatively high rates in central wards; this pattern was supported by a strong significant correlation between SIR's of males and females (0.71). There was a moderate negative correlation (-0.25) between SIR's of colorectal and esophageal cancer. In fact, eastern wards of Golestan province (Turkmen plain) which were high in esophageal cancer were among the lowest in colorectal cancer. Colorectal cancer is believed to be related to low levels of fiber consumption and high SES [39]. Accordingly, the fact that 55.9% of male and 57.7% of female inhabitants in the three local clusters of colorectal cancer lived in urban areas whereas only 42.3% of males and 42.2% of females outside the clusters lived in urban areas may be related to differences in diet and SES between urban and rural area.

#### Liver Cancer

Viral hepatitis is the major cause of liver disease and hepatocellular carcinoma. Up to 80% of liver cancers are believed to result from this viral infection which is the most important cause of cancer mortality worldwide after smoking. Data obtained from the Survey of Health and Disease in Iran indicted that the rate of hepatitis B virus (HBV) carriers varied between zero and 3.9% in different provinces of Iran with an average of 1.7% [52]. Hepatitis C virus (HCV) is another important risk factor and it has been shown that approximately 85% of individuals infected by HCV will develop chronic HCV infection [53]. In Iran, it seems that the prevalence of HCV in the general population is less than 1%, which is much lower than in most of the neighboring countries [54]. Currently, there are no data available about infection rates of HBV and HCV within the Caspian region.

#### Pancreas Cancer

Cancer of the pancreas had low prevalence and there was little evidence of spatial trends. The distribution of pancreas rates in the region was similar to a random pattern, and there was low correlation (0.09) between male and female rates.

## Conclusion

With media attention and an atmosphere of concern among the general population, it was difficult to dispassionately assess the strength of evidence for the existence of the hypothesized clusters of gastrointestinal tract cancer in northern Iran. The difficulty was compounded by the variety and complexity of available statistical methods for assessing the spatial variation of disease. Appropriate statistical methods for evaluation of geographic differences should i) provide global indices that summarize the spatial pattern of the disease, ii) smooth the observed map of disease for potential sampling variation while accounting for both region contiguity and region heterogeneity, and iii) search for possible disease clusters in a manner that adjust for the pre-selection bias and multiple testing effects. In this paper we used Moran's I and Geary's C as global autocorrelation indexes, a hierarchical Bayesian model for smoothing the cancer rates, and spatial scan statistics for cluster detection.

When a cluster of high incidence cannot be dismissed as a chance occurrence as is the case with many of the findings in this paper, we need to ask what may be the underlying causal mechanism. It is most natural to look first at some of the known or hypothesized risk factors. We have demonstrated that several cancer sites have significant regional variation in the Caspian region. An explanation for this spatial variation, however, requires further study, especially concerning the possible impact of environmental factors. Ecologic studies of the kind described here provide a relatively inexpensive way of examining regional variation in health in large populations. The effects of environmental factors can also be addressed by access to existing data sets. However, these studies involve interpretational problems arising from the aggregation of data and potential sources of bias such as variation in the size of the regional populations, migration, and disease latency [55,56].

With its large population, interesting regional pattern of GI cancer incidence as demonstrated here, differences in climate, life style, ethnic mix and variation in recognized risk factors, the Caspian region warrants further study into the relationship between environment and GI cancer.

## Competing interests

The authors declare that they have no competing interests.

## Authors' contributions

MoM and MaM designed and conducted the study. MaM was responsible for the data collection process and issues related to data quality. NK, ZH and FA assisted in designing and conducting the study. MoM performed the statistical analysis. MK supervised the study scientifically, and was involved in designing the study. MoM, WR and NK wrote the first draft of the manuscript to which all authors subsequently contributed. All authors read and revised the manuscript for important intellectual content and approved the final manuscript.

## Pre-publication history

The pre-publication history for this paper can be accessed here:


